# Potential value of a rapid syndromic multiplex PCR for the diagnosis of native and prosthetic joint infections: a real-world evidence study

**DOI:** 10.5194/jbji-9-87-2024

**Published:** 2024-02-28

**Authors:** Stéphanie Pascual, Brooklyn Noble, Nusreen Ahmad-Saeed, Catherine Aldridge, Simone Ambretti, Sharon Amit, Rachel Annett, Shaan Ashk O'Shea, Anna Maria Barbui, Gavin Barlow, Lucinda Barrett, Mario Berth, Alessandro Bondi, Nicola Boran, Sara E. Boyd, Catarina Chaves, Martin Clauss, Peter Davies, Ileana T. Dianzo-Delgado, Jaime Esteban, Stefan Fuchs, Lennart Friis-Hansen, Daniel Goldenberger, Andrej Kraševac Glaser, Juha O. Groonroos, Ines Hoffmann, Tomer Hoffmann, Harriet Hughes, Marina Ivanova, Peter Jezek, Gwennan Jones, Zeynep Ceren Karahan, Cornelia Lass-Flörl, Frédéric Laurent, Laura Leach, Matilde Lee Horsbøll Pedersen, Caroline Loiez, Maureen Lynch, Robert J. Maloney, Martin Marsh, Olivia Milburn, Shanine Mitchell, Luke S. P. Moore, Lynn Moffat, Marianna Murdjeva, Michael E. Murphy, Deepa Nayar, Giacomo Nigrisoli, Fionnuala O'Sullivan, Büşra Öz, Teresa Peach, Christina Petridou, Mojgan Prinz, Mitja Rak, Niamh Reidy, Gian Maria Rossolini, Anne-Laure Roux, Patricia Ruiz-Garbajosa, Kordo Saeed, Llanos Salar-Vidal, Carlos Salas Venero, Mathyruban Selvaratnam, Eric Senneville, Peter Starzengruber, Ben Talbot, Vanessa Taylor, Rihard Trebše, Deborah Wearmouth, Birgit Willinger, Marjan Wouthuyzen-Bakker, Brianne Couturier, Florence Allantaz

**Affiliations:** 1 bioMérieux, Marcy l'Etoile, France; 2 bioMérieux, Salt Lake City, USA; 3 University Hospital Southampton NHS Foundation Trust, Southampton, United Kingdom; 4 Newcastle Upon Tyne Hospitals NHS Foundation Trust, Newcastle Upon Tyne, United Kingdom; 5 S. Orsola Bologna, Microbiology Unit, IRCCS Azienda Ospedaliero-Universitaria di Bologna, Bologna, Italy; 6 Sheba Medical Center, Ramat Gan, Israel; 7 University Hospital of Wales, Cardiff, Wales, United Kingdom; 8 San Giovanni Battista, Department of Public Health and Pediatrics Microbiology and Virology Unit, Città della Salute e della Scienza, Turin, Italy; 9 Hull University Teaching Hospitals NHS Trust, Hull, United Kingdom; 10 Oxford University Hospitals (OUH), Oxford, United Kingdom; 11 AZ Alma, Eeklo, Belgium; 12 Department of Public Health and Pediatrics, University of Turin, Turin, Italy; 13 Mater Misericordiae University Hospital, Dublin, Ireland; 14 Chelsea and Westminster NHS Foundation Trust, London, United Kingdom; 15 Centro Hospitalar e Universitário de Coimbra, Coimbra, Portugal; 16 University Hospital Basel, Basel, Switzerland; 17 NHS Greater Glasgow and Clyde, Glasgow Royal Infirmary, University of Glasgow, Glasgow, United Kingdom; 18 Servicio de Microbiología, Hospital Universitario Ramón y Cajal and Instituto Ramón y Cajal de Investigación Sanitaria (IRYCIS), Madrid, Spain; 19 Dept. of Clinical Microbiology, IIS-Fundación Jiménez Díaz, CIBERINFEC-CIBER de Enfermedades Infecciosas, Madrid, Spain; 20 Institute of Hygiene and Medical Microbiology Medizinische Universität Innsbruck, Innsbruck, Austria; 21 Copenhagen University Hospital, Bispebjerg, Copenhagen, Denmark; 22 Dept. Clinical Microbiology at Rigshospitalet, Copenhagen, Denmark; 23 NZOLH Maribor, Maribor, Slovenia; 24 Varsinais-Suomen sairaanhoitopiiri, Loimaa, Finland; 25 MVZ Labor Dr. Reising-Ackermann und Kollegen, Limbach Leipzig, Germany; 26 East Tallinn Central Hospital, Tallin, Estonia; 27 Regional Hospital Příbram, Příbram, Czech Republic; 28 Ankara University School of Medicine Department of Medical Microbiology, Ankara, Türkiye; 29 Hospices Civils de Lyon, Lyon, France; 30 Centre Hospitalier Universitaire de Lille, Lille, France; 31 University Hospital “St George”, Plovdiv, Bulgaria; 32 Hampshire Hospitals NHS Foundation Trust, Winchester, UK; 33 Krankenhaus Göttlicher Heiland, Vienna, Austria; 34 Koper lab, Orthopedic Hospital Valdoltra, Valdoltra, Slovenia; 35 Careggi University Hospital, Florence, Italy; 36 Hôpital Ambroise Paré, APHP, Boulogne-Billancourt, France; 37 CIBER de Enfermedades Infecciosas, Instituto de Salud Carlos III. Servicio de Microbiología, Hospital Universitario Ramón y Cajal and Instituto Ramón y Cajal de Investigación Sanitaria (IRYCIS), Madrid, Spain; 38 Hospital Universitario Marqués de Valdecilla, Santander, Spain; 39 Allgemeines Krankenhaus Wien, Vienna, Austria; 40 University Medical Center Groningen, Groningen, the Netherlands

## Abstract

**Introduction**: The BIOFIRE Joint Infection (JI) Panel is a diagnostic tool that uses multiplex-PCR testing to detect microorganisms in synovial fluid specimens from patients suspected of having septic arthritis (SA) on native joints or prosthetic joint infections (PJIs). **Methods**: A study was conducted across 34 clinical sites in 19 European and Middle Eastern countries from March 2021 to June 2022 to assess the effectiveness of the BIOFIRE JI Panel. **Results**: A total of 1527 samples were collected from patients suspected of SA or PJI, with an overall agreement of 88.4 % and 85 % respectively between the JI Panel and synovial fluid cultures (SFCs). The JI Panel detected more positive samples and microorganisms than SFC, with a notable difference on *Staphylococcus aureus*, *Streptococcus* species, *Enterococcus faecalis*, *Kingella kingae*, *Neisseria gonorrhoeae*, and anaerobic bacteria. The study found that the BIOFIRE JI Panel has a high utility in the real-world clinical setting for suspected SA and PJI, providing diagnostic results in approximately 1 h. The user experience was positive, implying a potential benefit of rapidity of results' turnover in optimising patient management strategies. **Conclusion**: The study suggests that the BIOFIRE JI Panel could potentially optimise patient management and antimicrobial therapy, thus highlighting its importance in the clinical setting.

## 1 Introduction

The incidence of septic arthritis is 4–10 per 100 000 individuals per year, a figure which increases to 30–60 per 100 000 individuals for patients having underlying joint disease or a prosthetic joint (Geirsson et al., 2008; Weston et al., 1999; Favero et al., 2008; Smith et al., 2006; He et al., 2023; Patel, 2023). Younger children and older adults are the most vulnerable groups (Roerdink et al., 2019). The prevalence of prosthetic joint infections (PJIs) ranges from 2.0 %–2.4 % after primary interventions, a figure which increases to 20 % in patients undergoing revision procedures (Signore et al., 2019). About 60 %–70 % of PJIs occur during the first 2 years post-surgery, and nearly 50 % of all revision arthroplasties are due to PJI, contributing to a 1 %–3 % annual mortality (Postler et al., 2018).

Septic arthritis (SA) on native joints is usually aetiologically monomicrobial; the pathogen profile is relatively homogenous across patient populations; and it is associated with acute clinical manifestations, with classic signs and symptoms of swelling, pain, and redness at the infected site. Conversely, PJIs may be aetiologically mono- or poly-microbial and are commonly classified according to the time of presentation from implantation of the prosthesis (Zimmerli et al., 2004; Benito et al., 2019).

A number of fastidious pathogens which are more difficult to grow in culture can also be responsible for SA and PJI, including *Kingella kingae*, a common cause of SA in younger children (Hunter et al., 2022; Yagupsky, 2022), and *Neisseria gonorrhoeae*, an important cause of septic arthritis in newborns and sexually active individuals (Kleiman and Lamb, 1973; Moussiegt et al., 2022). Moreover, in an extensive review conducted by Tande and Patel (2014) including data from over 6700 specimens, the authors demonstrated that *Staphylococcus aureus* was the most common pathogen in early-onset PJI. These outcomes were consistent across other prominent publications for PJIs (Benito et al., 2019; Peel et al., 2012) and SA (Horowitz et al., 2011; He et al., 2023). When acute haematogenous cases were analysed, *Staphylococcus aureus* alone was identified in 68 % of samples (Stefánsdóttir et al., 2009). Thereafter, the most common pathogens are *Streptococcus* species, *Enterococcus* species, and gram-negative bacilli (Tande and Patel, 2014; Stefánsdóttir et al., 2009; Wouthuyzen-Bakker et al., 2019). Most notably, polymicrobial infections contribute to more than 31 % of early infections and 15 % for all time-period infections (Tande and Patel, 2014).

Traditional culturing of pathogens for a definitive microbiological diagnosis of joint infections has several limitations. Aside from limitations related to the turnaround time, traditional cultures are associated with a substantial false negative rate (Schulz et al., 2021), most notably related to pre-analytical issues (e.g. SA patients previously treated with antibiotics have a higher risk of false negative results with culture methods. Moreover, the probability of having false-negative results of PJI cultures increases with late compared with early post-surgical period infections and ranges from 6.5 %–17 % (Stefánsdóttir et al., 2009; Aggarwal et al., 2014). In a review of 64 manuscripts, the average sensitivity and specificity were, 65.6 % and 94.4 % respectively, whereas for molecular diagnosis, sensitivity and specificity were 74.1 % and 95.2 % respectively (Cozzi Lepri et al., 2019). In another study, among 543 cases of SA, only 40 % had positive synovial fluid cultures (McBride et al., 2020).

Remarkably, molecular methods such as PCR are often unavailable in hospitals and may also take 1–2 d to obtain a diagnosis due to turnaround time. Finally, cultures are much more likely to miss polymicrobial infections (Schulz et al., 2021).

A missed or delayed diagnosis can have a tremendous impact on patients and put high financial strain on our healthcare budget. Moreover, this can result in extended rehabilitation trajectories, permanent disabilities, and psychological consequences. It is known to increase morbidity and mortality. Also, prolonged administration of inappropriate antimicrobials can result in antimicrobial resistance, addressed by the WHO as one of the main global public health threats we are facing in our society.

Molecular diagnostic methods are currently being explored in various settings, and their benefits in proving critical information about the nature of the causative organisms to optimise targeted therapy is evident (Kullar et al., 2023). The BIOFIRE^®^ Joint Infection (JI) Panel, a rapid syndromic multiplex-PCR assay (hereafter the JI Panel), is an in vitro diagnostic assay which detects 31 bacterial and yeast nucleic acids and selects eight antimicrobial resistance genes most involved in joint infections. The JI Panel has 91.7 % sensitivity and 99.8 % specificity. The fully integrated all-in-one system required 2 min of hands-on time and 0.2 mL of synovial fluid and provides diagnostic results within approximately 1 h (bioMérieux, 2022). In recent studies, positive percent agreement and negative percent agreement for the JI Panel and routine culture for on-panel organisms on SA and PJIs were 91.6 % and 93.0 % (Saeed et al., 2022), and the comparison of JI Panel with culture on SA and acute PJI demonstrated a sensitivity of 80.6 % and a specificity of 100.0 % for the JI Panel (Schoenmakers et al., 2023).

The purpose of this study was to evaluate the agreement between the JI Panel and the synovial fluid culture and assess the user experience with the JI Panel in a multi-national site study of real-world settings.

## 2 Materials and methods

A total of 30 to 60 JI Panels were provided to 34 clinical sites across 19 countries in Europe and the Middle East. Instructions were provided to utilise the JI Panel per labelled indication in addition to the standard of care in patients suspected of having SA or PJI using leftover synovial fluids. The JI Panel includes 29 bacterial targets, 2 yeasts, and 8 resistance markers (details of the microorganisms are in Tables 2 and 3). The JI Panel tests were intended for investigational use only, and no data provided by the JI Panel were used for clinical decision-making. To capture the real-world data with the least amount of bias, the study allows the standards of care to vary (e.g. disease epidemiology, patient management methods, and facility capacities) across clinical sites. Data on the JI Panel results and their synovial fluid culture counterparts performed per standard of care in each centre were collected prospectively by individual investigators in each centre during approximately 16 months, from February 2021 to June 2022. Subsequently, the sites were asked to comment on whether the JI Panel results would have had an impact on patient management, in order to evaluate the user experience and possible perspectives in utilising the JI Panel in real-life use.

Only descriptive analyses were done for the data interpretation. Overall agreement was calculated at sample level, based on results obtained with synovial fluid routine culture and JI Panel for each sample; a positive result means that at least one microorganism was detected by the method. Then, the detection results by organism were assessed on if both or only one of the methods detected the microorganism. The final diagnosis of SA or PJI was not considered in this calculation. Ethical aspects, such as required approval and patient consent forms, were handled on a case-per-case basis depending on the site and country requirements. Only suspected cases of SA and PJI were included. In addition, all specimens in which cultures only grew organisms missing from the JI Panel menu were excluded for the on-panel analysis results.

## 3 Results

There were 1527 synovial fluid analysed across the 34 participating sites, including 873 (57 %) specimens for SA and 398 (26 %) in PJI. The sites reported use of the JI Panel most commonly in patients over the age of 56 years. The distribution of joint types was the following: knee (54 %), hip (16 %), shoulder (5 %), elbow (4 %), spine (2 %), ankle (2 %), and wrist (1 %).

Table 1 presents the patient age groups, joint infection types, and involved joints.

**Table 1:** Distribution of age, joint infection origin, and type.

Evaluable	Percent of samples
parameters	( n=1527 samples)
Age of patient	
≤18 years old	6 % (87)
19–55 years old	23 % (357)
≥56 years old	59 % (898)
Unknown	12 % (185)
Joint infection type	
Native	57 % (873)
Prosthetic	26 % (398)
Unknown	17 % (256)
Joint type	
Knee	54 % (826)
Hip	16 % (251)
Shoulder	5 % (79)
Elbow	4 % (57)
Spine	2 % (34)
Ankle	2 % (31)
Wrist	1 % (13)
Unknown	16 % (236)

### 3.1 Native joint infection microorganism distribution

#### 3.1.1 Overall agreement between synovial fluid cultures and JI Panel results at specimen level

The comparison by specimen between the JI Panel and synovial fluid cultures (SFCs) led to an overall agreement of 88.4 %. There were 147 specimens found to be positive (detection of at least one microorganism) by both SFC and the JI Panel, and 641 specimens were found to be negative by both SFC and the JI Panel. There were 73 specimens found to be positive only by the JI Panel and 12 specimens found to be positive only by SFC.

#### 3.1.2 Comparison of microorganism and resistance marker detections obtained by synovial fluid cultures and JI Panel

For each microorganism and resistance marker, Table 2 provides detections obtained with both SFC and JI Panel, detections only obtained with SFC, and detections only obtained with JI Panel. Table 2 also provides the total numbers of microorganisms and resistance markers detected by each method.

The most common microorganism identified was *Staphylococcus aureus*, followed by *Streptococcus* and *Enterococcus* species. Out of 26 *Streptococcus* spp. detected, 13 were only detected by the JI Panel, while SFCs were negative, whereas SFC detected *Streptococcus* spp. in three samples in which the JI Panel was negative. Out of 17 gram-positive anaerobes detected, the JI Panel detected 15 which were not detected by SFC, whereas both methods were positive in only two cases. Out of 17 detections of *Enterococcus* species, SFC detected 1 which was not detected by the JI Panel, and the JI Panel detected 7 which were not detected by SFC. Moreover, from a total of 117 *Staphylococcus aureus*, 26 were detected by the JI Panel and not by SFC, whereas 7 were detected by the JI Panel and not by SFC. In addition, out of 9 *Streptococcus pneumoniae*, 7 were detected by the JI Panel and not by SFC. Notably, the JI Panel detected 7 *Neisseria gonorrhoeae* and 3 *Kingella kingae* that SFC did not detect.

**Figure 1:**
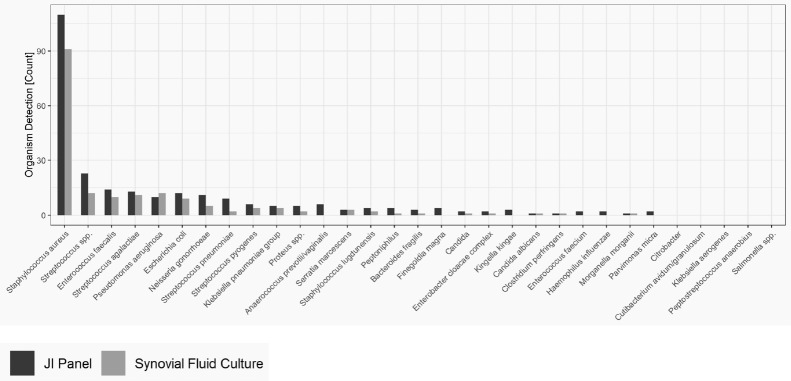
Distribution of microorganisms in native joint infections (on-panel).

Figure 1 provides the overall distribution by microorganism for SFC and the JI Panel. For all microorganisms, the detection rate is higher with the JI Panel than with SFC except for *Pseudomonas aeruginosa*.

In terms of antimicrobial resistance gene detection, out of 12 methicillin resistance genes associated with *Staphylococcus aureus* (*mecA/C*/MREJ), 6 were only detected by the JI Panel, 2 only by antimicrobial susceptibility testing following SFC, and 4 by both methods. JI Panel also detected resistance genes of CTX-M (2 associated with *Klebsiella pneumoniae*), NDM, OXA-48-like, and VIM (1 associated with *Serratia marcescens*).

Only microorganisms and resistance markers included in the JI Panel menu are reported in Table 2 and Fig. 1.

Regarding “off-panel” microorganisms, i.e. microorganisms not covered by the JI Panel and detected by SFC, the most common microorganisms identified were *Staphylococcus epidermidis* (6 %), followed by *Staphylococcus capitis* (4 %), *Bacillus* species (2 %), and *Cutibacterium acnes* (2 %).

**Table 2:** Detection results per microorganism and resistance genes for SFC and JI Panel in native joints (on-panel).

			Number	Number	Number	Number	Number	Number
			SFC ( + )	SFC ( - )	SFC ( + )	total ( + )	JI ( + )	SFC ( + )
			JI ( + )	JI ( + )	JI ( - )			
Gram-positive bacteria	Aerobes	*Enterococcus faecalis*	9	5	1	15	14	10
		*Enterococcus faecium*	0	2	0	2	2	0
		*Staphylococcus aureus*	84	26	7	117	110	91
		*Staphylococcus lugdunensis*	2	2	0	4	4	2
		*Streptococcus *spp.	10	13	3	26	23	13
		*Streptococcus agalactiae*	9	3	1	14	13	11
		*Streptococcus pneumoniae*	2	7	0	9	9	2
		*Streptococcus pyogenes*	3	3	1	7	6	4
	Anaerobes	*Anaerococcus prevotii/vaginalis*	0	6	0	6	6	0
		*Clostridium perfringens*	1	0	0	1	1	1
		*Cutibacterium avidum/granulosum*	0	0	0	0	0	0
		*Finegoldia magna*	0	4	0	4	4	0
		*Parvimonas micra*	0	2	0	2	2	0
		*Peptoniphilus*	1	3	0	4	4	1
		*Peptostreptococcus anaerobius*	0	0	0	0	0	0
Gram-negative bacteria	Aerobes	*Citrobacter *spp.	0	0	0	0	0	0
		*Enterobacter cloacae * complex	0	2	1	3	2	1
		*Escherichia coli*	9	3	0	12	12	9
		*Haemophilus influenzae*	0	2	0	2	2	0
		*Kingella kingae*	0	3	0	3	3	0
		*Klebsiella aerogenes*	0	0	0	0	0	0
		*Klebsiella pneumoniae *Group	4	1	0	5	5	4
		*Morganella morganii*	0	1	1	2	1	1
		*Neisseria gonorrhoeae*	4	7	1	12	11	5
		*Proteus* spp.	2	3	0	5	5	2
		*Pseudomonas aeruginosa*	9	1	3	13	10	12
		*Salmonella *spp.	0	0	0	0	0	0
		*Serratia marcescens*	1	2	2	5	3	3
	Anaerobes	*Bacteroides fragilis*	1	2	0	3	3	1
Yeasts		*Candida* spp.	0	1	0	1	1	0
		*Candida albicans*	1	0	0	1	1	1
Resistance markers	Gram-	*mecA/C and MREJ*	4	6	2	12	10	6
	positive	*van A/B*	0	0	0	0	0	0
	Gram-negative	CTX-M	0	2	0	2	2	0
		IMP	0	0	0	0	0	0
		KPC	0	0	0	0	0	0
		NDM	0	1	0	1	1	0
		OXA-48-like	0	1	0	1	1	0
		VIM	0	1	0	1	1	0

Abbreviations are as follows: JI – Joint Infection Panel, SFC – synovial fluid culture.

### 3.2 Prosthetic joint infection microorganism distribution

#### 3.2.1 Overall agreement between synovial fluid cultures and JI Panel results at specimen level

The comparison by specimen between the JI Panel and SFC led to an overall agreement of 85.7 %. There were 138 specimens found to be positive (detection of at least one microorganism) by both the JI Panel and SFC and 210 specimens found to be negative by both the JI Panel and SFC. There were 38 specimens found to be positive only by the JI Panel and 12 specimens found to be positive only by SFC.

#### 3.2.2 Comparison of microorganism and resistance marker detections obtained by synovial fluid cultures and JI Panel

For each microorganism and resistance marker, Table 3 provides detections obtained with both SFC and the JI Panel, detections only obtained with SFC, and detections only obtained with the JI Panel. Then, Table 2 also provides the total numbers of microorganisms and resistance markers detected by each method.

As obtained for SA, for PJI the most common microorganism identified was *Staphylococcus aureus*, followed by *Streptococcus* and *Enterococcus* species. Out of 74 *Staphylococcus aureus*, 14 were detected by the JI Panel only. Out of 21 *Enterococcus* species detections, 9 were detected by the JI Panel only. Similarly, out of 5 *Finegoldia magna* detections, 4 were only detected by the JI Panel. Out of 14 *Klebsiella* species detections, 8 were detected by JI Panel and not by SFC.

**Figure 2:**
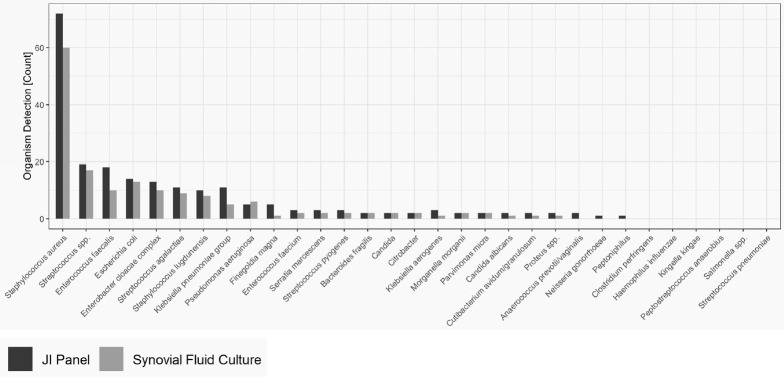
Distribution of microorganisms in prosthetic joint infections (on-panel).

Figure 2 provides the overall distribution by microorganism for SFC and JI Panel. For all microorganisms, the detection rate is higher with the JI Panel than with SFC except for *Pseudomonas aeruginosa*.

In terms of antimicrobial resistance gene detection, out of nine methicillin resistance genes associated with *Staphylococcus aureus* (*mecA/C*/MREJ), four were only detected by the JI Panel and five by both methods. The JI Panel also detected one resistance gene of *vanA/B* associated with *Enterococcus faecium* and CTX-M, NDM, OXA-48-like, and VIM resistance gene associated with gram-negative bacteria.

Only microorganisms and resistance markers included in the JI Panel menu are reported in Table 3 and Fig. 2.

Off-panel microorganisms for patients with PJI included similarly *Staphylococcus epidermidis* as the most prevalent (nearly 12 %) followed by *Bacillus* species (1.5 %), *Corynebacterium* species (1.5 %), *Cutibacterium acnes*, and *Staphylococcus capitis* (1 %).

**Table 3:** Detection results per microorganism and resistance genes for SFC and JI Panel in prosthetic joint infections (on-panel).

			Number	Number	Number	Number	Number	Number
			SFC ( + )	SFC ( - )	SFC ( + )	total ( + )	JI ( + )	SFC ( + )
			JI ( + )	JI ( + )	JI ( - )			
Gram-positive bacteria	Aerobes	*Enterococcus faecalis*	10	8	0	18	18	10
		*Enterococcus faecium*	2	1	0	3	3	2
		*Staphylococcus aureus*	58	14	2	74	72	60
		*Staphylococcus lugdunensis*	8	2	0	10	10	8
		*Streptococcus* spp.	15	4	3^a^	22	19	18
		*Streptococcus agalactiae*	9	2	0	11	11	9
		*Streptococcus pneumoniae*	0	0	0	0	0	0
		*Streptococcus pyogenes*	2	1	0	3	3	2
	Anaerobes	*Anaerococcus prevotii/vaginalis*	0	2	0	2	2	0
		*Clostridium perfringens*	0	0	0	0	0	0
		*Cutibacterium avidum/granulosum*	1	1	0	2	2	1
		*Finegoldia magna*	1	4	0	5	5	1
		*Parvimonas micra*	1	1	1	3	2	2
		*Peptoniphilus*	0	1	0	1	1	0
		*Peptostreptococcus anaerobius*	0	0	0	0	0	0
Gram-negative bacteria	Aerobes	*Citrobacter* spp.	1	1	1	3	2	2
		*Enterobacter cloacae* complex	9	4	1	14	13	10
		*Escherichia coli*	11	3	2	16	14	13
		*Haemophilus influenzae*	0	0	0	0	0	0
		*Kingella kingae*	0	0	0	0	0	0
		*Klebsiella aerogenes*	1	2	0	3	3	1
		*Klebsiella pneumoniae *group	5	6	0	11	11	5
		*Morganella morganii*	2	0	0	2	2	2
		*Neisseria gonorrhoeae*	0	1	0	1	1	0
		*Proteus* spp.	1	1	0	2	2	1
		*Pseudomonas aeruginosa*	3	2	3	8	5	6
		*Salmonella* spp.	0	0	0	0	0	0
		*Serratia marcescens*	2	1	0	3	3	2
	Anaerobes	*Bacteroides fragilis*	2	0	0	2	2	2
Yeasts		*Candida* spp.	0	0	1^b^	1	0	1
		*Candida albicans*	1	1>^b^	0	2	2	1
Resistance markers	Gram-	*mecA/C and MREJ*	5	4	0	9	9	5
	positive	*van A/B*	1	1	0	2	2	1
	Gram-negative	CTX-M	3	3	0	6	6	3
		IMP	0	0	0	0	0	0
		KPC	0	0	0	0	0	0
		NDM	0	4	0	4	4	0
		OXA-48-like	0	4	0	4	4	0
		VIM	0	2	0	2	2	0

Abbreviations are as follows: JI – Joint Infection Panel, SFC – synovial fluid culture. ^a^ One identified as *Streptococcus pyogenes* by JI Panel. ^b^ *Candida* spp. by SFC identified as *Candida albicans* by JI Panel.

### 3.3 User experience

The user experience was divided into the perceived experience by positive and negative detections by the JI Panel per SA and PJI. Approximately half of the utilisers provided their feedback on the potential benefits of the JI Panel. Among responders using the JI Panel for SA, 64 % reported that the JI Panel results would have modified the patient management when the panel detected a positive outcome (i.e. successfully detected a pathogen), and among responders using the JI Panel for PJI, 70 % reported that the JI Panel results would have modified the patient management when the panel detected a positive outcome. Furthermore, among responders using the JI Panel for SA, 31 % reported that the JI Panel results could have modified the patient management when the panel did not detect a positive outcome (i.e. no pathogen was detected by the panel), and among responders using the panel for PJI, 20 % reported that the JI Panel results could have modified the patient management when the panel did not detect a positive outcome.

## 4 Conclusions

Delay in diagnosis and inadequate antibiotic therapy in septic joints including PJI can lead to worse outcomes, and therefore enhancing microbiological diagnostic yield is an important factor in the management and outcome of septic arthritis (Costales and Butler-Wu, 2018).

We report the findings for a multi-national evaluation of the JI Panel in comparison with SFC for synovial fluid (native and PJI) samples to consider its potential impact on the management of joint infections in various laboratory settings.

At specimen level, the overall agreement between the two methods was high for both SA (88.4 %) and PJI (85.7 %), with more specimens found to be positive by the JI Panel than synovial fluid cultures. SFC is the current reference method but seems to fail to identify some microorganisms leading to a lower diagnostic yield than the JI Panel. Even if there were no discrepant analyses at the specimen level, these results are in accordance with recent publications, demonstrating that the positivity rate obtained by SFC is lower than the one obtained with the JI Panel (Saeed et al., 2022; Esteban et al., 2023).

The most frequent microorganisms identified in both types of infections were *Staphylococcus aureus*, *Streptococcus*, and *Enterococcus* species. The JI Panel found many additional detections of these three microorganisms, which could potentially lead to more appropriate antimicrobial treatment, a crucial element in tackling antimicrobial resistance, and better patient outcome. Additionally, the panel detected more anaerobic organisms which can be generally difficult to grow, or they can easily perish during suboptimal sample processing. More than 30 anaerobic microorganisms such as *Anaerococcus prevotii/vaginalis*, *Finegoldia magna*, *Peptoniphilus*, and *Parvimonas micra* were only detected by the JI Panel and not by SFC. While these microorganisms are less prevalent based on culture epidemiology, their detection by the JI Panel will potentially change the nature of the epidemiology of SA and PJI and possible antibiotics guidance; however we need more studies to confirm this impact. The JI Panel also identified some microorganisms that were overlooked by SFC, such as *Neisseria gonorrhoeae* and *Streptococcus pneumoniae*, which might not routinely be suspected by clinicians.

Early diagnosis of *Neisseria gonorrhoeae* septic arthritis is not only important for the patient but also for contact tracing as well checking other potential sexually transmitted diseases.

Regarding paediatric patients, *Kingella kingae* is the most prevalent pathogen for children under 5 years old. The JI Panel was able to detect three cases of *Kingella kingae* in paediatric SA samples that were missed by SFC as they are difficult to grow organisms.

Confirming the findings from Saeed et al. (2022), accurate identification in children with septic arthritis is critical to avoid cartilage damage and could also support an adjusted antimicrobial prescription with an earlier switch of route of administration and facilitate discharge (Alcobendas et al., 2023).

Additionally, it is important to note that the JI Panel's ability to quickly detect certain common antimicrobial resistance genes may optimise antimicrobial therapy which will impact antimicrobial stewardship, especially in regions with a high prevalence of drug resistant pathogens, as well as impact infection prevention, isolation for cases of methicillin-resistant *S. aureus* (MRSA), extended spectrum beta-lactamase producers (ESBL), and vancomycin-resistant enterococci (VRE). The JI Panel detected more resistance genes overall, especially for *Staphylococcus aureus* with methicillin resistance genes.

The study has limitations, including its retrospective nature; interlaboratory variations of SFC; and sites performing the study at different time points, with criteria for native SA or PJI that could have potentially varied from site to site based on suspicion of infection. Furthermore, as a non-interventional study, we have no prior information about antibiotic exposure and the impact of this on culture. Finally, we did not assess impact on antimicrobial management and infection prevention when a resistance gene was detected by the panel. Additionally, it is important to mention that a certain proportion of organisms identified by SFC were not detected by the JI Panel as the panel does not cover those specific organisms and not because of any limitations in the accuracy of detection, such as coagulase negative *Staphylococci* (apart from *S. lugdunensis*) and *Cutibacterium acnes*.

More studies such as controlled or interventional studies are needed to better evaluate the clinical impact in terms of antimicrobial resistance, surgical management, and long-term patient-reported outcomes.

Despite these limitations, the JI Panel detected more microorganisms and more resistance genes in both SA and PJI compared to synovial fluid cultures and applicable methods for resistance identification. Even if the study did not directly compare the turnaround times of SFC and the JI Panel, as per recent publications, the JI Panel, with a 1 h turnaround time, provided a faster time to results compared to a Gram stain and SFC, which can take up to 4 h and 14 d respectively (Azad et al., 2022; Berinson et al., 2023; Hoffman et al., 2023). Given the high precision of diagnosis with the JI Panel relative to SFC and the ability to quickly detect microorganisms and resistance markers, in 1 h, the JI Panel may yield a higher number of correctly identified pathogens in both septic arthritis in native joints and prosthetic joint infections, possibly leading to an optimised antimicrobial stewardship, as delayed appropriate therapy could increase the time of antibiotic therapy (Balada-Llasat et al., 2022). Hence, patients could also benefit from an earlier antibiotic de-escalation and/or switch of antibiotic (Berinson et al., 2023). This could also support the surgical strategy (Indelli et al., 2023) and appropriate patient management for better patient outcomes.

The JI Panel must always be used alongside synovial fluid culture for patients with a suspicion of SA or PJI, and the final diagnosis should follow dedicated guidelines. Preferences should not be given to a specific joint since there is no significant difference.

In this real-world evidence study, the JI Panel was provided to clinical sites across several countries to further understand its utility as assessed by the final users. The impact on patient management observed on positive samples is correlated with results obtained when culture as having a fast and accurate result could improve patient management. User experience needs to be further evaluated through surveys and testimonials to gather more informative data on its clinical utility.

In summary, the JI Panel had increased yield for on-panel organisms compared to synovial fluid cultures and with rapid turnaround times, demonstrating a potential adjuvant to standard cultures with more clinical impact on patient management.

## Supplement

10.5194/jbji-9-87-2024-supplement The supplement related to this article is available online at: https://doi.org/10.5194/jbji-9-87-2024-supplement.

## Data Availability

No data sets were used in this article.
