# Supplementary material for: Potential value of a rapid syndromic multiplex PCR for the diagnosis of native and prosthetic joint infections: a real-world evidence study
Source: J Bone Jt Infect. 2024 Feb 28;9(1):87–97. doi: 10.5194/jbji-9-87-2024 (PMC11002912; doi:10.5194/jbji-9-87-2024)
Supplement: The supplement related to this article is available online at: https://doi.org/10.5194/jbji-9-87-2024-supplement. [file jbji-9-87-supplement.pdf]

Supplement of J. Bone Joint Infect., 9, 87–97, 2024
https://doi.org/10.5194/jbji-9-87-2024-supplement
© Author(s) 2024. CC BY 4.0 License.

Supplement of

Potential value of a rapid syndromic multiplex PCR for the diagnosis of native and prosthetic joint infections: a real-world evidence study

Stéphanie Pascual et al.

Correspondence to: Stéphanie Pascual (stephanie.pascual@biomerieux.com)

The copyright of individual parts of the supplement might differ from the article licence.

BIOFIRE® JOINT INFECTION PANEL

GRAM POSITIVE BACTERIA

- **Aerobes**
- *Enterococcus faecalis*
- *Enterococcus faecium*
- *Staphylococcus aureus*
- *Staphylococcus lugdunensis*
- *Streptococcus* spp.
- *Streptococcus agalactiae*
- *Streptococcus pneumoniae*
- *Streptococcus pyogenes*

- **Anaerobes**
- *Anaerococcus prevotii/vaginalis*
- *Clostridium perfringens*
- *Cutibacterium avidum/granulosum*
- *Fingoldia magna*
- *Parvimonas micra*
- *Peptoniphilus*
- *Peptostreptococcus anaerobius*

GRAM NEGATIVE BACTERIA

- **Aerobes**
- *Citrobacter*
- *Enterobacter cloacae* complex
- *Escherichia coli*
- *Haemophilus influenzae*
- *Kingella kingae*
- *Klebsiella aerogenes*
- *Klebsiella pneumoniae* group
- *Morganella morganii*
- *Neisseria gonorrhoeae*
- *Proteus* spp.
- *Pseudomonas aeruginosa*
- *Salmonella* spp.
- *Serratia marcescens*

- **Anaerobes**
- *Bacteroides fragilis*

YEAST

- *Candida* spp.
- *Candida albicans*

GRAM POSITIVE RESISTANCE MARKERS

Methicillin Resistance

- *mecA/C* and MREJ (MRSA)

Staphylococcus aureus

Vancomycin Resistance

- *van A/B*

Enterococcus species

GRAM NEGATIVE RESISTANCE MARKERS

ESBL

- CTX-M

Carbapenemases

- IMP
- KPC
- NDM
- OXA-48-like
- VIM

Citrobacter
Enterobacter cloacae complex
K. aerogenes
K. pneumoniae group
M. morganii
Proteus spp
Salmonella
S. marcescens
P. Aeruginosa (except OXA-48-like)
